# Availability, Price, and Quality of Fruits and Vegetables in 12 Rural Montana Counties, 2014

**DOI:** 10.5888/pcd12.150158

**Published:** 2015-08-13

**Authors:** Carmen Byker Shanks, Selena Ahmed, Teresa Smith, Bailey Houghtaling, Mica Jenkins, Miranda Margetts, Daniel Schultz, Lacy Stephens

**Affiliations:** Author Affiliations: Selena Ahmed, Bailey Houghtaling, Mica Jenkins, Miranda Margetts, Daniel Schultz, Lacy Stephens, Montana State University, Bozeman, Montana; Teresa Smith, Gretchen Swanson Center for Nutrition, Omaha, Nebraska.

## Abstract

We assessed the consumer food environment in rural areas by using the Nutrition Environment Measures Survey for Stores (NEMS–S) to measure the availability, price, and quality of fruits and vegetables. We randomly selected 20 grocery stores (17 rural, 3 urban) in 12 Montana counties using the 2013 US Department of Agriculture’s rural–urban continuum codes. We found significant differences in NEMS–S scores for quality of fruits and vegetables; of 6 possible points, the mean quality score was 4.5; of rural stores, the least rural stores had the highest mean quality scores (6.0). Intervention strategies should aim to increase fruit and vegetable quality in rural areas.

## Objective

Rural populations are disproportionately affected by obesity and its associated chronic diseases (1). Access to healthy food is key in promoting intake of nutrient-dense foods that prevent nutrition-related chronic disease and obesity (2). Food environments with accessible and affordable healthful foods support healthful individual food choices and consumption (3). Research on food and store quality in the rural food environment is limited (4). A recent systematic review of the consumer food store environment found 3 times as many audits of urban environments (n = 39) as rural environments (n = 13); it also found the Nutrition Environment Measures Survey for Stores (NEMS–S) to be most frequently used to assess food availability (5). Of the 13 audits of the rural consumer food environment, only 8 used the NEMS–S. The objective of this study was to assess the consumer food environment in rural areas in Montana by using the NEMS–S to measure the availability, price, and quality of fruits and vegetables (5).

## Methods

This observational study of grocery stores in rural Montana towns was conducted from January to November 2014. NEMS–S was used to assess the availability, price, and quality of fruits and vegetables. Development and testing of the measurement tool is described elsewhere (6). The study was exempt from review.

Study sites were randomly selected on the basis of the 2013 US Department of Agriculture’s rural–urban continuum codes (RUCCs) (7). RUCCs range from 1 through 10: ranges 1 through 3 are classified as metro (urban; counties in metro areas; population ≥250,000), and 4 through 10 as nonmetro (rural; counties not in metro areas; population <250,000). No counties in the United States are classified as 10. We selected sites with a RUCC classification of 6 or higher (population <20,000).

Rural counties were randomly selected from a master list of Montana RUCCs by using a random number generator. Random selection of sites continued until at least 3 counties were identified for each RUCC classification 6 through 9. One urban control county was randomly selected.

The largest town by population size was systematically selected in each county. When a county was selected more than once, the next largest town by population size was selected. In each town, the largest grocer was selected (only 1 rural town selected had more than 1 grocer). Given the density of large grocers of the same size in the urban control county (Missoula County), we selected 3 urban stores.

The sample consisted of 20 stores in 17 towns in 12 counties. Six grocery stores were selected in RUCC 6, three grocery stores in RUCC 7, 4 grocery stores in RUCC 8, 4 grocery stores in RUCC 9. The rural counties were Choteau, Gallatin, Glacier, Jefferson, Lake, Madison, Meagher, Mineral, Pondera, Sanders, Teton, and Wheatland.

We calculated averages for total NEMS–S score (54 possible points), availability score (30 possible points), price score (18 possible points), and quality score (6 possible points) for fruits and vegetables. SAS version 9.2 (SAS Institute Inc) was used for statistical analysis. We used analyses of covariance to examine differences in county data (*P* < .05) and the Bonferroni correction to detect significant differences in pairwise comparisons (*P* < .01). Overall *P* values for differences in NEMS–S scores by 2013 RUCC (by county location of store) were obtained by using the Kruskal–Wallis test (7). Significance was set at a 2-sided α level of .05. A sensitivity analysis was conducted by using the Fisher exact test to determine significant differences in scores by county rurality.

## Results

We found significant differences in sociodemographic characteristics by county (Table 1). One in 5 residents (19%) was aged 65 years or older, 84% were non-Hispanic white, 90% had at least a high school degree, and 20% were living below the poverty level. The average household consisted of 2.4 members. Half of the stores (50%) were located on an Indian reservation, and most stores (88%) accepted the Supplemental Nutrition Assistance Program.

**Table 1:** Characteristics of County Rural Subgroups in Montana (n = 20), Study on Availability, Price, and Quality of Fruits and Vegetables, 2014

Characteristic (Year)	All Counties Combined	*P* Value	Stratified by 2013 Rural Urban Continuum Code (RUCC)^a^				
			RUCC 3	RUCC 6	RUCC 7	RUCC 8	RUCC 9
Population change (2010–2013), %	1	.03	2	1	2	0	1
Aged ≥65 (2013), %	19	<.001	13^b^	19	14^b^	23^c^	23^c^
Non-Hispanic white (2013), %	90	<.001	92^b^	66^c^	90^b^	88^b^	94^b^
High school graduates aged ≥25 y (2008–2012), %	84	.003	94^b^	90	84^c^	89	90
No. of persons per household (2008–2012), mean (SD)	2.4 (0.3)	.006	2.3 (0)	2.3 (0)^b^	2.9 (0.3)^c^	2.4 (0.2)	2.4 (0.3)
Population living below poverty level (2008–2012), %	20	<.001	17	23^b^	25^b^	19	12^c^

Abbreviation: SD, standard deviation.

a RUCCs range from 1 through 10: ranges 1 through 3 are classified as metro (urban; counties in metro areas; population ≥250,000), and 4 through 10 as nonmetro (rural; counties not in metro areas; population <250,000).

b, c Values within a row that do not share a common superscripted letter (b, c) are significantly different (*P *< .01), whereas values that do share a common superscripted letter are not significantly different.

For fruits and vegetable in all 20 stores, the (mean) NEMS–S total score was 23.8; availability score, 17.1; price score, 2.9; and quality score, 4.5. NEMS–S total scores, availability scores, and price scores did not differ by county rurality, but quality scores did (Table 2). Of stores in rural counties, stores in the least rural area (RUCC 6) had the highest quality scores (mean, 6.0).

**Table 2:** Analysis of Variance of NEMS–S Scores for Fruits and Vegetables by County Rurality Measured by 2013 Rural Urban Continuum Code (n = 20), Study on Availability, Price, and Quality of Fruits and Vegetables, Montana, 2014

RUCC^a^	NEMS–S Score, Mean (SD)			
	Total^b^	Availability^c^	Price^d^	Quality^e^
3	28.7 (7.4)	22.7 (2.5)	3.7 (4.7)	5.7 (0.6)
6	28.2 (5.5)	18.5 (5.2)	3.7 (2.5)	6.0 (0)
7	14.7 (17.5)	9.0 (11.5)	2.0 (3.5)	3.7 (3.2)
8	25.5 (4.7)	19.5 (2.6)	2.5 (1.3)	3.5 (2.6)
9	21.8 (2.2)	15.8 (4.1)	2.5 (3.0)	3.5 (1.3)

Abbreviation: NEMS–S, Nutrition Environment Measures Survey for Stores; RUCC, rural–urban continuum code; SD, standard deviation.

a RUCCs range from 1 through 10: ranges 1 through 3 are classified as metro (urban; counties in metro areas; population ≥250,000), and 4 through 10 as nonmetro (rural; counties not in metro areas; population <250,000).

b Of 54 possible points; *P* = .35, Kruskal–Wallis test for overall differences in NEMS–S scores by RUCC.

c Of 30 possible points; *P* = .17, Kruskal–Wallis test for overall differences in NEMS–S scores by RUCC.

d Of 18 possible points; *P* = .87, Kruskal–Wallis test for overall differences in NEMS–S scores by RUCC.

e Of 6 possible points; *P* = .03, Kruskal–Wallis test for overall differences in NEMS–S scores by RUCC.

## Discussion

Research exploring rural food access has used limited parameters, such as number of food stores within a certain radius (4); few studies have used NEMS–S (5). This study used NEMS–S and demonstrated that availability and price of fruits and vegetables did not differ by rurality. However, quality was significantly lower in more rural locations.

Rural residents are less likely than their urban counterparts to consume 5 servings of fruits and vegetables per day (8), are at higher risk for diabetes and heart disease, and are more likely to be obese (9–11). Montana adults consume a daily median of 1.0 fruit serving and 1.6 vegetable servings (12); rural adults are less likely than nonrural adults in Montana to consume 5 or more daily servings of fruits and vegetables (8). Fruit and vegetable consumption is associated with lower rates of chronic disease (13).

Broad study findings provide some insight on factors that influence the quality of produce (14); food stores are less available in rural than in urban areas (15), and physical infrastructure is a major barrier to food access in rural areas (16). Future research should focus on finding solutions for improving the quality of fruits and vegetables and its impact on purchases and consumption. We hypothesize that limited infrastructure for food distribution (eg, roads, storage, frequency of delivery) in rural areas poses obstacles to maintaining high-quality produce. Additionally, poor-quality produce may drive rural consumers from the produce aisle to processed foods.

This study was limited to rural locations in Montana; application of results may be inappropriate in other locations. Because of the extensive driving time between study sites and weather and road conditions in Montana, data collection took place during 11 months; this long data collection period may have affected NEMS–S scores. Also, rural residents might purchase fruits and vegetables from places other than the largest grocer in their town.

Findings indicate the need for research and intervention strategies that are tailored to rural areas, increase produce quality, improve dietary and health outcomes, and decrease health disparities.
